# Favipiravir treatment does not influence disease progression among adult patients hospitalized with moderate-to-severe COVID-19: a prospective, sequential cohort study from Hungary

**DOI:** 10.1007/s11357-021-00452-9

**Published:** 2021-09-03

**Authors:** Balint Gergely Szabo, Katalin Szidonia Lenart, Borisz Petrik, Zsofia Gaspar, Noemi Kiss-Dala, Janos Szlavik, Istvan Valyi-Nagy, Botond Lakatos, Saint Ladislaus COVID-19 Collaborative

**Affiliations:** 1 South Pest Central Hospital, National Institute of Hematology and Infectious Diseases, Saint Ladislaus Campus, H-1097 Albert Florian ut 5‑7., Budapest, Hungary; 2 Doctoral School of Clinical Medicine, Semmelweis University, H-1085 Ulloi ut 26, Budapest, Hungary; 3 Faculty of Medicine, Semmelweis University, H-1085 Ulloi ut 26, Budapest, Hungary

**Keywords:** SARS-CoV-2, COVID-19, Favipiravir, Antiviral

## Abstract

Data suggests that favipiravir (FVP) could be used against SARS-CoV-2. Our aim was to investigate the role of FVP in COVID-19 treatment. A prospective sequential cohort study was performed among adults hospitalized at our center between March and August 2020 with moderate-to-severe, PCR-confirmed COVID-19. For diagnosis and severity, ECDC and WHO definitions were utilized. Patients were screened for inclusion by a priori criteria and included in the FVP cohort if standard-of-care (SOC) + FVP or the non-FVP cohort if SOC ± other antivirals without FVP were administered for > 48 h from diagnosis. Treatment allocation was done per national guidelines, based on severity and drug availability. Primary endpoint was disease progression, a composite of 14-day all-cause death, need for mechanical ventilation, or immunomodulatory therapy. The impact of FVP exposure on disease progression was analyzed by binomial logistic regression. In all, 150 patients were included, 75 in each cohort. Disease progression (17/75, 22.7% vs. 10/75, 13.3%, *p* = 0.13), 14-day all-cause death (9/75, 12.0% vs. 10/75, 13.3%, *p* = 0.8), and need for mechanical ventilation (8/75, 10.7% vs. 4/75, 5.3%, *p* = 0.22) were similar, while immunomodulatory therapies were required more frequently among patients receiving FVP (10/75, 13.3% vs. 1/75, 1.3%, *p* < 0.01). The use of favipiravir was not retained as a protective factor against disease progression in multivatiate analysis. Time to antiviral therapy from PCR positivity, disease severity, need for oxygen supportation, and ICU admittance rates did not differ statistically between cohorts. In this study, favipiravir did not seem to positively affect disease progression.

## Introduction

As the pandemic caused by severe acute respiratory syndrome coronavirus-2 (SARS-CoV-2) is ongoing, investigators are searching for therapeutic strategies against coronavirus disease-19 (COVID-19). As of April 2021, the number of antiviral drugs proven to inhibit viral replication in vivo is low [1]. Favipiravir (FVP) is a pyrazinecarboxamide derivative, licensed for influenza treatment in Japan since 2014. Literature data suggested that favipiravir might be useful for COVID-19 treatment. During in vitro studies, FVP demonstrated inhibitory activity against SARS-CoV-2. Also, early clinical experience from two trials recruiting adult inpatients documented favorable therapeutic responses with FVP in terms of recovery and viral clearance [2–5]. These results highlighted the need for further data concerning the place of favipiravir among COVID-19 treatment strategies. In Hungary, favipiravir was one of the drugs approved for clinical use as an experimental medication during the first wave on May 15, 2020. Our goal was to assess the effect of favipiravir on the clinical course of moderate-to-severe COVID-19 among inpatients.

## Methods

### Study design and settings

A prospective sequential cohort study was performed among consecutive adult (age ≥ 18 years) patients diagnosed and hospitalized with COVID-19 between March and August 2020 at our center. The first COVID-19 case was confirmed in March 4 in Hungary. Our center is a national-level referral institution of COVID-19 with > 150 dedicated beds. The study was in accordance with the Helsinki Declaration and national ethical standards. The institutional review board of our center approved the study protocol. During the first wave of the COVID-19 pandemic, a randomized study design was not ethically feasible. Approval for the use of off-label antiviral drugs was granted by the National Institute of Pharmacy and Nutrition and the institutional review board. Informed consent was obtained from each patient.

### Patient eligibility and inclusion

Patients hospitalized at our center during the study period with COVID-19, confirmed by respiratory SARS-CoV-2 polymerase chain reaction (PCR), were eligible for inclusion, irrespective of COVID-19 severity. To overcome selectional bias, all patients were screened for inclusion during daily real-time ward and intensive care unit (ICU) visits by our COVID-19 team, composed of attending physicians. Selection was done by using a priori inclusion/exclusion criteria after diagnosis establishment. Inclusion criteria are as follows: (1) moderate-to-severe COVID-19, confirmed by PCR, and (2) administration of standard-of-care (SOC) or any antiviral treatment exposures for > 48 h after diagnosis. Exclusion criteria are as follows: (1) the patient was intubated, died, or discharged within ≤ 48 h after diagnosis; (2) received SOC or any antiviral treatment exposures for ≤ 48 h after diagnosis; (3) received any other antiviral medication (e.g., against HIV, HBV, or HCV) before diagnosis; or (4) patient data was inaccessible through hospital electronic databases.

Included patients were grouped in a 1:1 proportion into two sequential “before/after” cohorts, according to favipiravir availability: FVP cohort consisted of patients receiving SOC + FVP (after availability), non-favipiravir (non-FVP) cohort included patients who were administered SOC ± other antiviral medications (before availability). Possible non-FVP antiviral medications at study design: chloroquine/hydroxychloroquine, lopinavir/ritonavir, or remdesivir (see below for details).

### Data collection

A database was established for the study aim by manual data extraction from hospital electronic records and written charts. Anonymized data were transferred to a standardized case report form. Data collected are as follows: (1) age and gender; (2) comorbidities; (3) ICU admission, length of stay (LOS), and ICU LOS; (4) baseline clinical parameters (symptom onset, COVID-19 severity, oxygen demand, peripheral oxygen saturation, acute respiratory distress syndrome [ARDS], cytokine storm, acute respiratory failure); (5) baseline laboratory parameters (absolute neutrophil granulocyte and lymphocyte counts, CRP, procalcitonin, serum ferritin, high-sensitivity cardiac troponin-I, serum interleukin-6 [IL-6], serum creatinine, LDH, and d-dimer); (6) baseline microbiological and radiological parameters (blood cultures and chest computed tomography [CT]); (7) antimicrobial, immunomodulatory therapies, and supportive care during hospitalization; (8) outcomes. Baseline characteristics were established at COVID-19 diagnosis. Variables with ≥ 5% of missing measurements were omitted from analysis.

### Diagnostic and therapeutic strategies at our center during the first wave

At our center, we followed the *European Centre for Disease Prevention and Control* (ECDC) COVID-19 case definition for diagnosis ascertainment: a clinically suspicious case (usual symptoms: fever, dyspnea, cough) was confirmed if a respiratory specimen was positive for SARS-CoV-2 nucleic acid by PCR [6]. Respiratory specimens were taken by trained nurses with nasopharyngeal sampling in spontaneously breathing patients or blind bronchoalveolar lavage in ventilated patients. Disease severity was determined by the World Health Organization (WHO) criteria [7]. Disease onset was the first day of patient-reported typical symptom apperance, or day of first PCR positivity, if symptoms could not be reported. The day of first SARS-CoV-2 PCR positivity was given as COVID-19 diagnosis day. Acute respiratory failure and ARDS were defined by 2012 Berlin criteria. Cytokine storm was diagnosed by a compatible case presentation (persistent fevers for ≥ 72 h, deteriorating hypoxaemia) with serum ferritin ≥ 600 µg/l, serum IL-6 ≥ 3 × and LDH level ≥ 1 × above the upper limit-of-normal, or a HScore of ≥ 250 [8–10]. COVID-19 patient care was facilitated by standardized and regularly updated in-house protocols since March 2020. Physical examination, laboratory studies, and arterial blood gas analyses were done daily. Chest X-ray and/or chest CT were executed on COVID-19 diagnosis day, and if clinical instability (newly onset dyspnea, chest pain, hypotension, altered mentation) was documented. Febrile patients had 2 sets of blood cultures taken. Fever was defined as a tympanal temperature ≥ 37.8 °C. Patient follow-up was done daily until death or hospital discharge. All microbiological diagnostics were performed at the microbiology laboratory of our center.

Anti-SARS-CoV-2 antiviral therapies were allocated per protocol according to COVID-19 disease severity in an open-label, non-randomized fashion. Treatment allocation was done in accordance with the “Hungarian Coronavirus Handbook,” and was affected by national drug availability [11]. Before favipiravir introduction, all inpatients with moderate-to-severe COVID-19 received other medications: chloroquine (1 g loading dose and 1 × 500 mg maintenance, 7 days minimum), hydroxychloroquine (2 × 400 mg loading dose and 2 × 200 mg maintenance, 5 days minimum), and lopinavir/ritonavir (200/50 mg in 2 × 2 capsules, 7 days minimum), depending on availability and contraindications. Although remdesivir was recommended in the protocol, it was not available in Hungary during the study period. All patients received favipiravir monotherapy after national distribution (2 × 1600 mg loading dose and 2 × 600 mg maintenance, 10 days minimum). Immunomodulatory drugs administered to patients with cytokine storm or critical COVID-19 were tocilizumab, ruxolitinib, baricitinib, intravenous immunoglobulin, convalescent plasmatherapy, or systemic corticosteroids. SOC included on-demand oxygen therapy, respiratory support, intravenous fluid replacement, antipyretics, antitussive, and bronchodilator drugs. All patients were given SOC independently from antiviral therapies. Empirical antibiotics according to *Infectious Diseases Society of America* (IDSA) community-acquired pneumonia guideline were initiated per decision of the attending physician, if clinical instability was documented and a bacterial cause could not be ruled out [12].

### Outcomes

Primary outcome was disease progression during COVID-19 treatment, a composite endpoint of any of the following: (1) 14-day all-cause death, (2) need for mechanical ventilation, (3) need for immunomodulatory therapy for COVID-19. Fourteen-day all-cause death was defined as exitus within 14 days from COVID-19 diagnosis during hospitalization. Need for mechanical ventilation was defined as endotracheal intubation in relation to COVID-19, per decision of an ICU crash team. Need for immunomodulatory therapy was defined if any immunomodulatory drug was initiated at any dose, excluding systemic corticosteroids started for alternative causes.

Secondary endpoints were 14-day all-cause mortality, need for mechanical ventilation, and need for immunomodulatory therapy (at hospital discharge or sooner). Analyses were done by comparing time intervals from COVID-19 diagnosis day to disease progression, all-cause mortality, mechanical ventilation, and immunomodulatory therapy.

### Statistical analysis

Continuous variables are expressed as median ± interquartile range (IQR). Categorical variables are expressed as absolute numbers (*n*) with relative percentages (%). Statistical comparisons were done with *Mann–Whitney U*-test or *Fisher*’s exact test. Normality was checked with *Shapiro–Wilk* test. For identification of independent risk factors associating with disease progression, uni- and multivariate binomial logistic regression was performed. Plausible parameters and those with a *p*-value ≤ 0.1 in univariate analysis were entered into forward-stepwise multivariate logistic regression (entry criterion: *p* = 0.05, removal criterion: *p* = 0.1). Maximal predictor number was estimated with the 1:10 rule-of-thumb; goodness-of-fit was tested by *Hosmer–Lemeshow* test. A two-tailed *p*-value < 0.05 determined statistical significance. Tests were calculated using IBM SPSS Statistics 23. For reporting, we adhered to *Strengthening the Reporting of Observational studies in Epidemiology* (STROBE) Statement [13].

## Results

### Baseline and clinical characteristics

In all, 150 patients were enrolled, 75 in both cohorts. Baseline and clinical characteristics are described in Table 1. Median age was 66.0 ± 12.4 years, with representation of older patients in the FVP cohort (71.5 ± 15.1 vs. 61.0 ± 21.5 years, *p* = 0.01). Genders and most comorbidities were equally distributed between cohorts, while chronic heart disease (36/75, 48.0% vs. 16/75, 21.3%, *p* < 0.01) and diabetes mellitus (18/75, 24.0% vs. 6/75, 8.0%, *p* = 0.01) were prevalent in the FVP cohort. At diagnosis, 35/75 (53.3%) and 41/75 (54.7%) patients had severe disease (*p* = 0.41); ARDS or cytokine storm was not documented. Need for oxygen supportation (27/75, 36.0% vs. 21/75, 28.5%, *p* = 0.29), and rates of ICU admission (12/75, 16.0% vs. 5/75, 6.7%, *p* = 0.07) did not differ statistically between cohorts during hospitalization. Bloodstream-infections were rare (1/75, 0.7%). Between cohorts, laboratory parameters were comparable, and chest CT positivity rate did not show statistical difference (54/64, 84.4% vs. 13/14, 92.8%, *p* = 0.67).

**Table 1:**
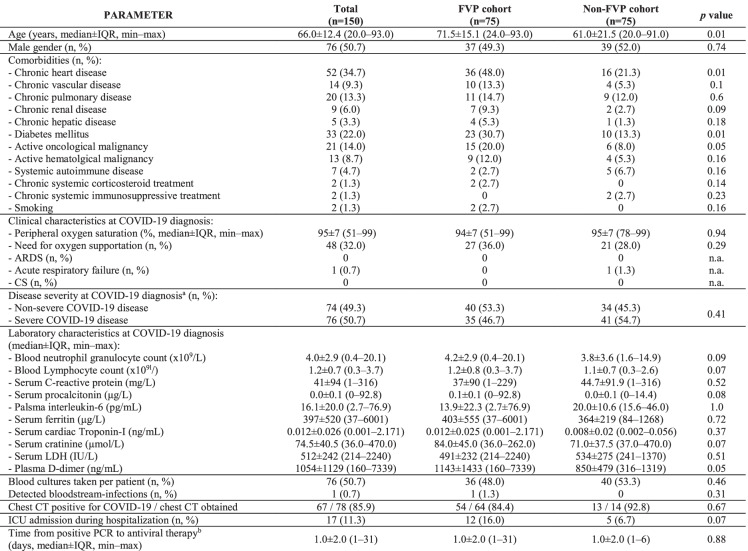
Demographic and clinical characteristics of adult COVID-19 patients included in the study, grouped by favipiravir exposure

*n.a.*, not applicable^a^Per the World Health Organization criteria^b^From first positive respiratory SARS-CoV-2 PCR sample

### Outcomes and therapeutic approaches

Outcomes and therapeutic approaches are detailed in Table 2. Disease progression showed no statistically significant difference between cohorts (17/75, 22.7% vs. 10/75, 13.3%, *p* = 0.13). Rates of 14-day all-cause mortality (9/75, 12.0% vs. 10/75, 13.3%, *p* = 0.8) and need for mechanical ventilation (8/75, 10.7% vs. 4/75, 5.3%, *p* = 0.22) were also similar. The need for any immunomodulatory therapy was higher in the FVP cohort (10/75, 13.3% vs. 1/75, 1.3%, *p* < 0.01). Also, both median time from diagnosis to disease progression (8.0 ± 9.0 days vs. 4.5 ± 9.8, *p* = 0.08) and to exitus (16.0 ± 14.0 days vs. 8.5 ± 10.3 days, *p* = 0.03) were longer among these patients. In the non-FVP cohort, patients usually received chloroquin or hydroxychloroquin. Frequently administered antibiotics were azithromycin (19/75, 25.3% vs. 44/75, 58.7%, *p* < 0.01) and ceftriaxon (13/75, 17.3% vs. 28/75, 37.3%, *p* < 0.01), while mostly tocilizumab was given to patients with cytokine storm (9/75, 12.0% vs. 1/75, 1.3%, *p* = 0.01). Supportive therapies detailed in Table 2 are required in statistically similar rates among cohorts (43/75, 57.3% vs. 46/75, 61.3%, *p* = 0.73). Favipiravir exposure was not retained as an independent protective factor in multivarite regression for disease progression (Table 3).

**Table 2:**
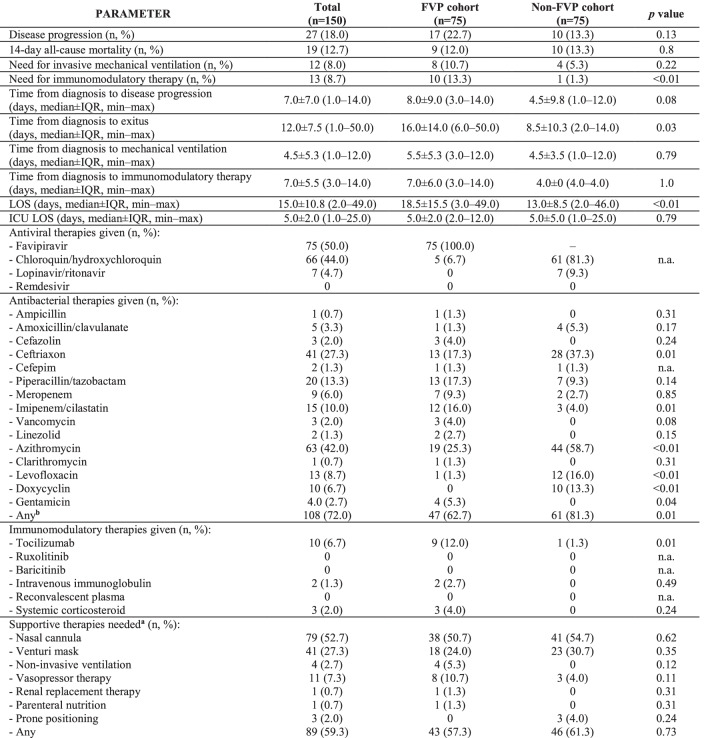
Outcomes and therapeutic approaches to adult COVID-19 patients included in the study, grouped by favipiravir administration

*n.a.*, not applicable^a^Excluding invasive mechanical ventilation^b^Alone or in combination

**Table 3:** Independent predictors of disease progression among adult COVID-19 patients included in the study, grouped by progression occurence

	Disease progression (*n* = 27)	No disease progression (*n* = 123)	Univariate analysis		Multivariate analysis	
			OR (95% CI)	*p* value	OR (95% CI)	*p* value
Age	74.0 ± 23.1 (41.0–91.0)	66.0 ± 12.9 (20.0–93.0)	1.04 (1.01–10.9)	0.01	–	
Male gender	12 (44.0)	64 (52.0)	0.74 (0.32–1.72)	0.47		
Time from disease onset to antiviral therapy	3.3 ± 7.8 (1–34)	3.5 ± 7.8 (1–34)	0.91 (0.81–1.01)	0.07*	n.a	
Chronic heart disease	18 (66.7)	34 (27.6)	5.81 (2.32–14.70)	< 0.01	4.27 (1.41–12.98)	0.01
Diabetes mellitus	10 (37.0)	23 (18.7)	2.69 (1.08–6.71)	0.03	–	
Need for oxygen supportation	16 (59.3)	32 (26.0)	4.13 (1.74–9.80)	0.01	–	
Severe COVID-19	26 (96.3)	50 (27.0)	38.5 (5.0–333.30)	< 0.01	21.28 (2.32–200.0)	< 0.01
Any supportive therapy needed	26 (96.3)	63 (51.2)	5.10 (1.82–14.30)	< 0.01	–	
Any antibacterial therapy needed	22 (81.5)	86 (69.9)	1.89 (0.67–5.37)	0.23		
Any antiviral therapy needed	25 (92.6)	114 (92.7)	1.01 (0.21–4.98)	0.98		
Treatment with favipiravir	17 (63.0)	58 (47.2)	1.91 (0.81–4.48)	0.14	–	

*n.a.*, not applicable

^*^The parameter was not included in the final model as co-linearity was not proven by the *Box-Tidwell* test with *Bonferroni*’s post hoc correction (*p* < 0.01)

## Discussion

### Present study

We performed a prospective sequential cohort study by enrolling 150 hospitalized adult patients with moderate-to-severe COVID-19, receiving either favipiravir or other antiviral medications with standard-of-care during the first wave of the SARS-CoV-2 pandemic in Hungary. There were no statistically significant differences in time to antiviral therapy initiation from PCR positivity, COVID-19 disease severity, need for oxygen supportation, and ICU admittance rates between cohorts. Disease progression, 14-day all-cause mortality, and need for invasive mechanical ventilation were unaffected by favipiravir exposure, compared to other antivirals. Suprisingly, there was higher demand for immunomodulatory therapies among patients receiving favipiravir. Finally, favipiravir was not proven as a protective factor against disease progression in multivariate analysis.

### Studies from the current literature

Pharmacological approaches and clinical studies describing favipiravir treatment strategies for COVID-19 patients were reported in the literature. Although favipiravir demonstrated good in vitro inhibitory activity against SARS-CoV-2, the optimal dose for COVID-19 treatment has yet to be determined, as recommendations are based on pharmacokinetic experiments and earlier clinical trials. Doses extrapolated from studies involving patients with other viral infections (influenza virus, Ebola virus) might be insufficient to maintain adequate serum concentrations, especially in critically ill patients [2, 14, 15]. Although a review found that favipiravir has a favorable safety profile concerning serious adverse events, the main side effects are hyperuricaemia, teratogenicity, and QTc prolongation. Establishment of long-term safety profile among COVID-19 patients needs more pharmacovigilance data [16].

Corcerning clinical data, clinical usefulness of favipiravir in COVID-19 may somewhat be limited. A prospective clinical trial randomizing 240 adult patients with clinically confirmed COVID-19 to conventional therapy and umifenovir or favipiravir reported that although favipiravir associated with shorter time to defervescence and diminishment of cough, the drug could not significantly improve 7-day clinical recovery rate as a primary endpoint. Furthermore, rates of noninvasive mechanical ventilation, supplementary oxygen demand, or all-cause mortality did not show differences between groups [4]. An open-label non-randomized study conducted by matching 35 microbiologically confirmed COVID-19 patients treated with favipiravir and 45 patients receiving lopinavir/ritonavir (all with interferon-alpha inhalation) within 1 week after symptom onset found statistically higher improvement rates in chest imaging and faster viral clearance among patients receiving favipiravir, but effects on disease progression or mortality were unreported [5]. A recently published phase II/III randomized trial enrolled patients with moderate COVID-19 within a median of 6.7 days from symptomp start, with a primary endpoint of SARS-CoV-2 elimination by day 10. On day 5, viral clearance was more prevalent on the favipiravir arm, but on day 10, this statistical difference diminished. Authors concluded that favipiravir appeared beneficial among moderately ill patients [17]. In small case series studies, favipiravir was administered with nafamostat mesylate or methylprednisolone for COVID-19 in different stages, but due to antiviral combination usage, the extent of favipiravir effect on clinical cure remains ambiguous [18–20]. In all, we think that our findings are reflected by current literature data, and the role for favipiravir in the treatment of adults with moderate-to-severe COVID-19 should probably be interpreted cautiously.

### Study limitations

Our study had several limitations. As knowledge about COVID-19 is changing at a rapid pace, treatment allocations described in the methods might have been lagging behind evidence despite our best efforts. National drug availability might have affected treatment allocation, while the decision between alternative antiviral agents in the non-FVP cohort might have been biased by contraindication(s). A randomized study design was not feasible during the first wave in Hungary due to ethical concerns. The number of included patients is relatively low; however, an exact a priori study size calculation was not feasible due to study design and consecutive enrollment. Although there are differences between cohorts concerning age and two comorbidities, we hypothesize that this might represent the temporal progression of the epidemic in Hungary, as younger people without comorbidities were mostly affected before FVP became widely available.

## Conclusion

Among adult patients hospitalized with modetare-to-severe COVID-19, an overall beneficial effect of favipiravir on disease progression could not be proven in this study. Further trial data are needed to elucidate the role of favipiravir in COVID-19 treatment.

## Data Availability

Anonymised data of patients are available from the corresponding author on reasonable request.

## Abbreviations

ARDS: Acute respiratory distress syndrome
COVID-19: Coronavirus disease-19
CRP: C-reactive protein
CT: Computed tomography
ECDC: European Centre for Disease Prevention and Control
EUCAST: European Committee on Antimicrobial Susceptibility Testing
FVP: Favipiravir
ICU: Intensive care unit
IQR: Interquartile region
LDH: Lactate dehydrogenase
LOS: Length of stay
PCR: Polymerase chain reaction
RCT: Randomized clinical trials
SARS-CoV-2: Severe acute respiratory syndrome coronavirus-2
SOC: Standard-of-care
STROBE: Strengthening the Reporting of Observational studies in Epidemiology
